# Neural Correlates of Reactive Aggression in Adult Attention-Deficit/Hyperactivity Disorder

**DOI:** 10.3389/fpsyt.2022.840095

**Published:** 2022-05-19

**Authors:** Babette Jakobi, Alejandro Arias-Vasquez, Erno Hermans, Priscilla Vlaming, Jan Buitelaar, Barbara Franke, Martine Hoogman, Daan van Rooij

**Affiliations:** ^1^Department of Human Genetics, Donders Institute for Brain, Cognition and Behavior, Radboud University Nijmegen Medical Center, Nijmegen, Netherlands; ^2^Centre for Cognitive Neuroimaging, Donders Institute for Brain Cognition and Behavior, Nijmegen, Netherlands; ^3^Department of Psychiatry, Donders Institute for Brain, Cognition and Behavior, Radboud University Nijmegen Medical Center, Nijmegen, Netherlands; ^4^Department of Cognitive Neuroscience, Donders Institute for Brain, Cognition and Behavior, Radboud University Nijmegen Medical Center, Nijmegen, Netherlands

**Keywords:** adult ADHD, emotion dysregulation, dynamic facial expressions, reactive aggression, task-based fMRI

## Abstract

Despite not being part of the core diagnostic criteria for attention-deficit/hyperactivity disorder (ADHD), emotion dysregulation is a highly prevalent and clinically important component of (adult) ADHD. Emotionally dysregulated behaviors such as reactive aggression have a significant impact on the functional outcome in ADHD. However, little is known about the mechanisms underlying reactive aggression in ADHD. In this study, we aimed to identify the neural correlates of reactive aggression as a measure of emotionally dysregulated behavior in adults with persistent ADHD during implicit emotion regulation processes. We analyzed associations of magnetic resonance imaging-based whole-brain activity during a dynamic facial expression task with levels of reactive aggression in 78 adults with and 78 adults without ADHD, and also investigated relationships of reactive aggression with symptoms and impairments. While participants with ADHD had higher reactive aggression scores than controls, the neural activation patterns of both groups to processing of emotional faces were similar. However, investigating the brain activities associated with reactive aggression in individuals with and without ADHD showed an interaction of diagnosis and reactive aggression scores. We found high levels of activity in the right insula, the hippocampus, and middle and superior frontal areas to be particularly associated with high reactive aggression scores within the ADHD group. Furthermore, the limbic activity was associated with more hyperactivity/impulsivity symptoms. These results suggest a partly differential mechanism associated with reactive aggression in ADHD as compared to controls. Emotional hyper-reactivity in the salience network as well as more effortful top–down regulation from the self-regulation network might contribute to emotionally dysregulated behavior as measured by reactive aggression.

## Introduction

Attention-deficit/hyperactivity disorder (ADHD) is a highly prevalent neurodevelopmental disorder (1), characterized by core symptoms of inattention and/or hyperactivity and impulsivity (2). Symptoms of ADHD persist into adulthood in up to 66% of affected individuals (1) and are commonly accompanied by emotion regulation problems (3). Even though symptoms of emotion dysregulation (ED) are prevalent in people with ADHD (with 24–50% in children and up to 70% in adults) and are an important predictor of ADHD symptoms (4), they have long been disregarded in diagnostic and therapeutic context (3).

An important expression of severe ED in ADHD is reactive aggression (5–9). Not only is aggressive behavior a frequent catalyst for diagnostic consultation (6), recent research reports that reactive aggression in ADHD remains significantly elevated after correction for comorbidities such as conduct disorder and oppositional defiant disorder (8, 10). Literature often distinguishes two types of aggressive behaviors. While proactive aggression links to instrumentalized and controlled aggressive behaviors, reactive aggression is a mirror of a dysregulated emotional response, e.g., to fear or anger (11). Reactive aggression can have a large impact on multiple dimensions of life. People with ADHD and co-occurring aggression show the most maladaptive strategies in emotion regulation and social decision making (12), often resulting in unstable dysfunctional relationships and families, peer rejection and victimization, functional impairments in school and later occupation, as well as an elevated risk of contact with criminality (6, 9, 10, 13). Reactive aggression has also consistently been linked to suicidal behaviors and attempts (9, 14). Abel et al. (9) reported that this elevated risk of suicidal behavior in reactive aggression is modulated by hyperactivity and impulsivity symptoms, irrespective of comorbidities such as depression.

Altered structural or functional maturation of several brain areas might point toward a neurodevelopmental link of ADHD with reactive aggression. Among small morphological differences in several areas, the structural alterations implicated in ADHD involve reduced volumes within the limbic system, e.g., the amygdala and the hippocampus (15), as well as differential cortical thickness in frontal and parietotemporal brain regions (16). Besides structural implications in ADHD, these regions also exhibit altered functional connectivity and altered activity profiles in ADHD [as reviewed by Rubia (17)]; they have been linked to altered emotional reactivity and memory [limbic system, orbito, and ventromedial frontal cortex (18, 19)] as well as executive functioning and attentional frontal and parietotemporal networks (20).

Emotional subprocesses are relevant for the emergence of reactive aggression and are frequently assessed using functional magnetic resonance imaging (fMRI) paradigms inducing an implicit or explicit emotional reaction to emotionally salient stimuli, e.g., the implicit processing of facial emotional expressions. FMRI studies in children and adolescents with ADHD have revealed patterns of elevated bottom-up emotional reactivity, reflected in altered activity in the amygdala, insula, ventral striatum, and the orbitofrontal cortex (OFC; 17, 21–24). Additionally, differences in the top–down modulation of emotional responses were found in tasks involving active emotion regulation. Differential activation of the amygdala or insula and hypo-connectivity of those structures to prefrontal structures, such as the ventrolateral PFC, the anterior cingulate cortex (ACC), and the temporoparietal junction (TPJ) have been observed in ADHD (25–27). Only few fMRI studies on face emotion processing were carried out in adults with ADHD, covering only small, remitted, or partially remitted samples and focusing on response inhibition and attention. The authors reported (subthreshold) activity- and connectivity differences in limbic and prefrontal circuit in an emotional go/no-go task (28) or hyperactivity in face-processing areas and differential connectivity to regions linked to attention in remitted adults in a dynamic facial expression task (29).

The above-mentioned brain regions altered in people with ADHD are overlapping broadly with the neural correlates of reactive aggression. While reactive aggressive behavior appears to be facilitated by activity of the limbic system and hypothalamus, prefrontal activity seems to indicate inhibition of such behaviors (30). Alia-Klein et al. (31) summarize the emergence of anger as a basis of reactive aggression to (1) reactivity of the salience network (dACC, Insula and limbic structures), influenced by (2) social cognition and self-referential processes in the mentalizing network (TPJ, SFG, posteriorCC, and dorsolateralPFC) and downregulated by (3) the self-regulation network (PFC, ACC, and IFG).

Hence, reactive aggression is associated with an imbalance of cognitive control (implemented in prefrontal areas) and hyper-reactivity to emotional stimuli of the limbic system and insula (8, 32, 33).

Despite the high impact on the quality of life, our understanding of the co-occurrence of ADHD with and reactive aggression and of the underlying mechanisms is limited. Neural circuits engaged in reactive aggression -as a severe form of ED- overlap with structurally and functionally implicated brain regions in ADHD and are linked together in functional neuroimaging studies on children and adolescents. However, research on the neural circuits or alterations during emotion processing underlying reactive aggression in adults with persistent ADHD is clearly underrepresented, does not cover implicit facial emotion processing nor integrate behavioral impairments such as ratings of reactive aggression.

This study aimed to identify the neural correlates of reactive aggression in adults with persistent ADHD during implicit emotion regulation processes. We acquired fMRI during a dynamic facial expression task (34, 35). We investigated the neural correlates of reactive aggression in adults with and without ADHD and analyzed the covariance of whole-brain activity with levels of reactive aggression scores from a questionnaire. We expected reactive aggression to be associated with altered neural activation within the emotion regulation network. Moreover, we aimed to identify subprocesses relevant for reactive aggression. We hypothesized more emotional reactivity, as reflected in hyperactivity of the limbic system and anterior insula, and/or more effortful or less cognitive control processes, as reflected in differential prefrontal activity to be relevant for the occurrence of higher reactive aggression in ADHD. We also explored the association of ADHD diagnosis and clinically relevant variables with the reactive aggression scores and *post hoc* correlations with the neural activity in areas implicated in reactive aggression in the fMRI analysis [insula, hippocampus, precentral gyrus, superior and middle frontal gyrus, middle temporal gyrus (MTG), and lingual gyrus].

## Materials and Methods

### Participants and Experimental Procedure

A total number of 83 adults with a confirmed diagnosis of ADHD and 79 healthy control subjects participated in this fMRI experiment. Participants were recruited *via* newspaper advertisements, patient organizations, and local sports clubs in and around Nijmegen, Netherlands. All of the participants provided written informed consent before participating in the study and received monetary compensation for their participation. The study was approved by the local medical ethical committee.

Participants were included in the ADHD group if they had been diagnosed with ADHD by a clinician. To confirm the diagnosis and assess previous and current symptoms in all participants, we conducted the Diagnostic Interview for Adult ADHD [DIVA 2.0; (36)]. The DIVA includes nine subscales for the symptoms of inattentiveness and hyperactivity/impulsivity and further assesses subjective impairment over life domains such as occupation, family and relationships, social contacts, hobbies and self-image in childhood as well as adulthood. Participants were included in the control group when the following criteria were met: absence of previous diagnoses of ADHD, of current neurological or psychiatric disorders and of first-degree family members with ADHD. Exclusion criteria for all participants comprised (1) an age younger than 18 or older than 60 years, (2) neurological disorders, (3) psychosis or substance abuse in the last 6 months, (4) current major depression, (5) psycho-pharmaceutical therapy other than stimulants, (6) impairments of hearing, seeing and sensorimotor abilities as well as (7) problems with understanding Dutch (to ensure that all of the participants understood the study protocol and the task instructions). The 40 participants with ADHD that received regular pharmacological treatment with stimulants, were asked to pause their medication intake 24 h prior to participation. Missing data of the reactive proactive aggression questionnaire of five participants from the ADHD and failed fMRI data preprocessing of one control subject resulted in a total sample of 78 participants with and 78 participants without ADHD. Both groups had comparable distributions of age, sex, IQ, and educational background (see Table 1 for a demographic description of the sample including the relevant questionnaire data).

**TABLE 1:** Demographic description of the sample.

Measure	Control group, *n* = 78	ADHD group, *n* = 78	Difference, *p*-value
Sex, percentage male participants	48.7%	43.6%	*0.596*
Age in years (SD)	34.2 (13.1)	34.1 (10.54)	*0.656*
Education (SD)	4.6 (1.6)	4.1 (1.6)	*0.019*
IQ (SD)	106.1 (13.6)*	108.7 (13.8)	*0.479*
**DIVA, mean number of symptoms**			
Attention symptoms in Adulthood (or current; SD)	0.79 (1.27)	7.32 (1.99)	*p* < 0.001***
Attention symptoms in childhood (SD)	0.49 (0.84)	7.23 (1.83)	*p* < 0.001***
Hyperactivity/Impulsivity adult (SD)	0.83 (1.37)	5.59 (2.24)	*p* < 0.001***
Hyperactivity/Impulsivity child (SD)	0.83 (1.36)	5.57 (2.65)	*p* < 0.001***
**DIVA, percentage of adults reporting impairment**			
Occupation	0%	74.3%	*p* < 0.001***
Relationship and family	0%	65.4%	*p* < 0.001***
Social contacts	0%	39.7%	*p* < 0.001***
Hobby	1.2%	53.8%	*p* < 0.001***
Self-image	0%	64.1%	*p* < 0.001***
**RPQ, mean score**			
Reactive aggression score (SD)	5.57 (3.31)	8.17 (4.05)	*p* < 0.001***
Proactive aggression score (SD)	1.42 (2.43)	1.90 (2.61)	0.242

Mean scores and standard deviations of age, highest achieved educational degree (measured on a scale of 1 to 8 in the Dutch education system), BMI, IQ score, number of present symptoms of inattention and hyperactivity/Impulsivity in childhood and adulthood and the percentage of subjects reporting impairments in occupation, relationship and family, social contacts, hobbies, and self-image from the DIVA. The bottom of the table shows the mean scores and standard deviation of the results from the RPQ, proactive aggression is excluded from further analysis. *The IQ estimate of one control subject was missing. Statistical testing was performed using the Mann–Whitney test as well as the Chi-squared test for distribution free comparisons of independent samples with a significance level of p = 0.001, marked by ***.

Participation was structured in two parts. The first part included the diagnostic screening for ADHD using the DIVA (36) and a short screening for comorbid psychiatric disorders following the Structured Clinical Interview for DSM, SCID-5. Demographic information was collected and IQ testing was performed using block-design and vocabulary subtests of the Wechsler Adult Intelligence Scale (37). In the second part, structural and functional magnetic resonance imaging (MRI) scans were acquired. After the visit, participants were asked to fill in questionnaires *via* an online platform, among others the Reactive-Proactive Aggression Questionnaire [RPQ; (11)]. The RPQ is a 23-item self-report questionnaire inquiring 11 example sentences of reactive and 12 of proactive aggression that are scored by the participant in a scale of never, sometimes and often. As reactive, but not proactive aggression is implicated in ED as well as ADHD, only the reactive subscale was used in the subsequent analyses. In the Supplementary Section “Analysis of Proactive Aggression,” Supplementary Table 2, we attached a linear regression on proactive aggression and ADHD.

To investigate implicit emotion processing during MRI, an adapted dynamic facial expression task was applied, showing faces morphing from a neutral face to an angry, fearful or happy facial expression in short clips of four frames. This task has proven to elicit activity in structures reflecting emotion processing, such as the amygdala (38). The stimuli were taken from a standardized set and consisted of 10 gray-scale clips per emotion, each represented by a different actor of male and female gender in equal distribution. During the experimental session, we presented 6 blocks per emotion with a duration of 22.5 s, which consisted of 50 trials, 5 repetitions for each of the 10 actors. The blocks were presented in counterbalanced order interleaved with 9 blocks showing a fixation cross. In one random trial of each block, a red dot was displayed on the forehead of the actor (see Supplementary Figure 1 in the section Supplementary Material). Participants were asked to press the button on a response box fixated on their leg as soon as the red dot appeared on the actor’s face, to sustain their attention while preserving passive processing of the emotional faces. The scanning time for this task was approximately 10 min.

### Image Acquisition

Magnetic resonance imaging scans were conducted using a 32-channel coil and a 3 Tesla Siemens Magnetom Prisma scanner (Siemens Trio, Erlangen, Germany). A T1-weighted MPRAGE sequence (TI = 1,100 ms, flip angle = 8°, TE = 3.03 ms, TR = 2300.0 ms, bandwidth = 130 Hz/Px) with 192 sagittal slices (slice thickness = 1.0 mm) was used for the structural scanning, providing whole-brain coverage. Functional blood oxygen level-dependent (BOLD) images were collected using a T2*-weighted echo-planar imaging (EPI) sequence (TR = 1,000 ms, TE = 34.0, flip angle = 60°, FOV = 210 mm, voxel size = 2 mm × 2 mm × 2 mm, 66 slices, interleaved acquisition, slice thickness = 2.00 mm). Preprocessing was performed in FSL FEAT. The first 5 images were discarded from further analysis. Mean framewise displacement of all participants was below the cutoff of 0.5 mm for 10% of the frames. After grand mean scaling and boundary based registration to the structural image and realignment as motion correction, a Gaussian filter of 5 mm kernel was applied to the images. Motion correction was performed using a dedicated independent component analysis based selection algorithm (39). Additionally, the average signal of white-matter and corticospinal fluid were subtracted from the data. For analyses on the group level, we normalized individual scans to Montreal Neurological Institute 152 standard space, 2 mm resolution.

### Analysis

#### Functional Magnetic Resonance Imaging Task Activation

Single subject fMRI analysis were performed in FSL FEAT (version 6.0.3) using a general linear model (GLM) with three regressors of interest modeling the onsets of happy, angry and fearful face blocks with a duration of 22.5 s as well two regressors of no interest modeling the trials where the red dot indicated the attention control task and the timing of the response as event markers with a duration of 0 s. As the task distracted from emotion processing, related BOLD activity and behavioral results of the task were excluded. Results were corrected for age and sex. All events were convolved with the canonical hemodynamic response function.

Three group level contrasts were defined by contrasting each emotional condition against the implicit baseline of the fixation blocks resulting in Happy > Fixation, Angry > Fixation, and Fear > Fixation images. We included the factors diagnosis in two groups and emotion in the three levels angry, fear and happy in a mixed factorial model and investigated the group effects for each emotion separately as well as for all conditions together (Emotion > Fixation). Results are reported at a cluster-level corrected significance threshold of *p* < 0.05.

We further investigated effects of sex on the brain activity during emotion processing.

#### Analysis of Functional Magnetic Resonance Imaging Task Activation and Reactive Aggression

To analyze the relationship between reactive aggressive scores with implicit emotion regulation and emotional reactivity, we included the contrast of all emotions versus the implicit baseline (Emotion > Fixation) as one summary measure per subject as well as the individual reactive aggression scores as a mean centered continuous covariate in the second level GLM. We were interested in the group specific correlates of reactive aggressive behavior, more specifically which brain regions would be relevant for higher reactive aggression scores in ADHD. Therefore we first investigated the interaction of diagnosis with reactive aggression, to see if there were group differences dependent on reactive aggression scores. Based on this interaction, we further looked at the effect in each group individually to find clusters associated with the co-occurance of higher reactive aggression within the ADHD group. To correct for multiple comparisons in this exploratory analysis, we applied a Monte-Carlo-simulation of 1,000 iterations for cluster extent correction on the uncorrected *t*-maps at *p* = 0.001 [(40, 41), this method is publicly accessible at https://drive.google.com/file/d/16HVUD-PZaEpwHoZE99YXDxhcuLawjW7O/] resulting in a cluster extent of 11 resampled voxels at a cluster extent threshold of *p* = 0.05 assuming a type 1 error of 0.01.

#### *Post hoc* Associations of Clinical Measures

To investigate the association between ADHD and the reactive aggression score of the RPQ, we used a linear regression analysis, modeling the Reactive Aggression scores by the binomial factor “diagnosis” (1 = ADHD group, 0 = control group). We included age and sex as covariates in the model. The analysis was conducted in R version 3.6.1.

To investigate the association of clinical outcome with aggression, we introduced DIVA subscales of adult symptoms (Hyperactivity/Impulsivity, Inattention) and impairments (Occupation, Relationships and family, Social contacts, Hobbies, and Self-image) as compounds to a linear model of reactive aggression scores. To assess clinical implications of our results, we analyzed the relationship of ADHD specific neural correlates of reactive aggression with the clinical expression of ADHD. We therefore analyzed correlations between symptoms and the activity in the right limbic system, the right precentral, inferior middle temporal and lingual gyri as well as the middle and superior frontal clusters showing positive covariance with elevated reactive aggression. Results are reported as Spearman’s ρ and Bonferroni-corrected for multiple correlations.

We additionally performed a sensitivity analysis to investigate potential influences of medication on reactive aggression, the number of hyperactivity and impulsivity symptoms and impairments measured with the DIVA as well as the brain activity associated with reactive aggression in the ADHD group, see the section Supplementary Analysis.

## Results

### Functional Magnetic Resonance Imaging Task Activation

For the whole-brain analysis of viewing all emotions compared to the implicit baseline, ADHD cases and controls did not show any differential activation patterns. Both diagnostic groups showed BOLD responses to emotional faces in broad clusters spanning several areas. These regions included temporoparietal, inferior, and superior frontal cortical areas as well as several subcortical areas including parts of the limbic system, see Supplementary Table 1 and Supplementary Figure 2. No significant differences were found either when analyzing the emotions separately. Comparisons were made at the FWE-corrected threshold of *p* = 0.05.

We furthermore found two clusters in the left superior frontal as well as left OFC associated with sex differences during emotion processing, see Supplementary Table 3.

### Neural Correlates of Reactive Aggression

#### Interaction of Reactive Aggression and Diagnostic Group

**FIGURE 1:**
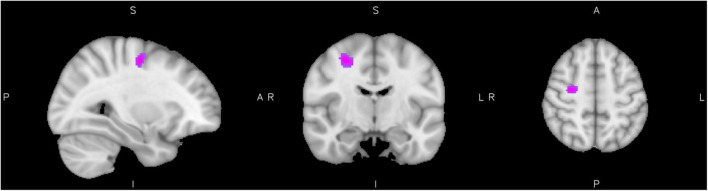
Interaction of diagnosis with reactive aggression. Results from the interaction of reactive aggression and diagnosis at the maximum of the cluster in the precentral gyrus (slices *x* = 30 left, *y* = −10 middle, and *z* = 52 right), cluster extent corrected for *p* = 0.05.

We found a significant interaction between ADHD diagnosis and reactive aggression scores in the activation of clusters assigned to the right precentral and postcentral gyri, superior parietal, middle temporal areas, lingual gyrus, and the caudate nucleus at a significance level of *p* = 0.05. Activity was higher for high reactive aggression scores in the ADHD group and lower for low reactive aggression scores in healthy controls. Figure 1 shows the cluster of significant interaction, for further information see Table 2. There were no clusters showing an inverse effect.

**TABLE 2:** Interaction of diagnosis and reactive aggression.

Cluster label	Voxels	*P*	Z-MAX	*X* (mm)	*Y* (mm)	*Z* (mm)
R Precentral gyrus	127	<0.05	4.97	30	−10	52
R Lingual gyrus	63	<0.05	3.68	16	−54	−6
R Superior parietal lobe	39	<0.05	3.84	30	−56	58
R Inferior/middle temporal gyrus	24	<0.05	3.64	58	−36	−16
R Postcentral gyrus	22	<0.05	3.86	18	−42	58
R Occipital Pole	11	<0.05	3.34	4	−86	32
L Caudate	11	<0.05	3.54	−6	8	10

*Results of the whole-brain analysis for the interaction of reactive aggression with diagnosis, cluster extent correction of 11 voxel for p = 00.05.*

#### Reactive Aggression in the Attention-Deficit/Hyperactivity Disorder Group

**FIGURE 2:**
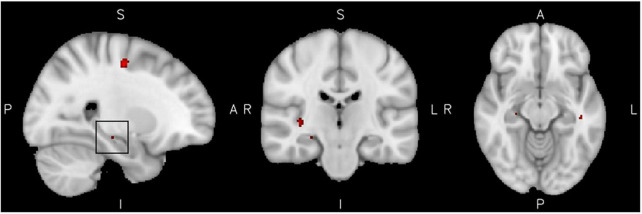
High reactive aggression in ADHD. Results from the reactive aggression analysis in the ADHD group at the maximum of the cluster of 14 voxel in the hippocampus, highlighted by a black square in the left figure (slices *x* = 26 left, *y* = −22 middle, and *z* = −12 right), at a cluster extent correction of *p* = 0.05.

The analysis of reactive aggression within the ADHD group showed significantly elevated activation levels of clusters including the precentral gyrus, cortical frontal and temporal areas, as well as subcortical structures such as the hippocampus. Furthermore two clusters within the right Amygdala [*p*_unc._ < 0.001, *xyz* = (22, −8, −8) and *p*_unc._ < 0.001 *xyz* = (28, −10, −1)] were activated, but did not exceed the threshold of 11 voxels each. The activity in all clusters was positively associated with higher reactive aggression scores. There was no significant effect of low reactive aggression, see Figure 2 and Table 3 for more information on the significant clusters.

**TABLE 3:** Neural correlates of reactive aggression.

Cluster label	Voxels	*P*	Z-MAX	*X* (mm)	*Y* (mm)	*Z* (mm)
**ADHD**						
R Precentral gyrus	83	<0.05	5.45	24	−12	50
L Middle frontal gyrus	42	<0.05	3.74	32	18	56
R Superior frontal gyrus	33	<0.05	3.78	6	32	62
R Inferior/middle temporal gyrus	33	<0.05	4.99	64	−32	−18
R Insula	20	<0.05	4.38	36	−22	2
R Lingual gyrus	18	<0.05	3.5	16	−70	−6
Not assigned	16	<0.05	4.27	−44	−26	−12
Not assigned	14	<0.05	3.82	30	2	28
R Hippocampus	14	<0.05	3.43	26	−22	−12
**Controls**						
L Midde/inferior temporal gyrus	240	<0.05	4.37	−62	−60	−4
R Superior parietal lobule	88	<0.05	3.82	28	−56	58
R Lingual gyrus	60	<0.05	3.77	18	−52	−4
R Precentral gyrus	24	<0.05	3.78	32	−10	56
R Superior Parietal Lobule	13	<0.05	3.62	28	−42	58

*Results of the whole-brain analysis for the analysis of reactive aggression within the ADHD group (top) and the control group (bottom) separately, cluster extent correction of 11 voxel for p = 0.05.*

#### Reactive Aggression in the Control Group

**FIGURE 3:**
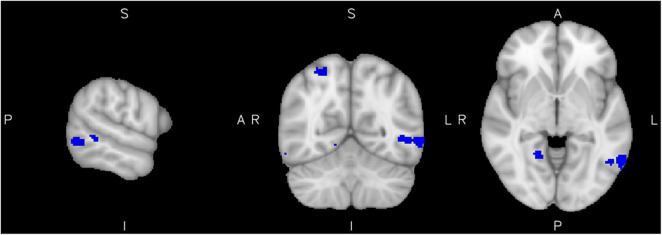
Low reactive aggression in the control group. Results from the reactive aggression analysis of the control group at the maximum of the cluster in the left middle temporal gyrus (slices *x* = −62 left, *y* = −60 middle, and *z* = −4 right), at a cluster extent correction of *p* = 0.05.

No positive relationship of reactive aggression with brain activity was found in the control group. However, we additionally investigated a potential inverse effect, e.g., negative associations of reactive aggression scores with the brain activity of healthy controls. We found a negative relationship of reactive aggression with the activity in a cluster in the left MTG, right superior parietal lobule as well as the right precentral and lingual gyri (Figure 3 and Table 3).

### *Post hoc* Associations of Clinical Measures

The linear regression analysis of reactive aggression modeled by diagnostic group revealed a significant association of the factor diagnosis with reactive aggression scores (*t* = 4.50; *p* < 0.001, *r*_standardized_ = 0.34). Age was not associated with reactive aggression, but male gender was (*t* = −3.72; *p* = 0.004, *r*_standardized_ = −0.28). Table 4 summarizes the results of the regression analysis.

**TABLE 4:** Regression of reactive aggression.

Regressors	Estimate	Standard error	*t*-value	*p*-value
ADHD diagnosis (y/n)	0.33	0.57	4.50	<0.001***
Age	0.03	0.02	0.40	0.69
Sex	–0.28	0.57	–3.72	<0.001***

Summary of regression analysis of reactive aggressive behavior, showing regression coefficients, standard errors, t- and p-values as well as levels of significance in codes from 0.001 as “***,” from 0.01 as “**,” or from 0.05 as “*” to the model.

We furthermore found associations between reactive aggression scores and the number of hyperactivity/impulsivity symptoms as well as self-reported impairment in the domains of “relationships and families” as well as “self-image” in adulthood from the DIVA in the regression analysis of the DIVA subscales, see Table 5.

**TABLE 5:** Associations of clinical measures with reactive aggression.

Regressors	Estimate	Standard error	*t*-value	*p*-value
Hyperactivity/Impulsivity	0.28	0.17	2.11	0.036*
Inattention	0.09	0.14	0.71	0.479
Occupation	0.34	0.93	0.11	0.917
Relationship and family	6.41	0.89	2.07	0.041*
Social contacts	2.03	0.81	0.71	0.479
Hobby	–2.17	0.79	–0.78	0.436
Self-image	–6.59	0.93	–2.02	0.045*

Summary of regression analysis of reactive aggressive behavior, showing regression coefficients, standard errors, t- and p-values as well as levels of significance in codes from 0.001 as “***,**”** from 0.01 as “**,” or from 0.05 as “*” to the model.

**FIGURE 4:**
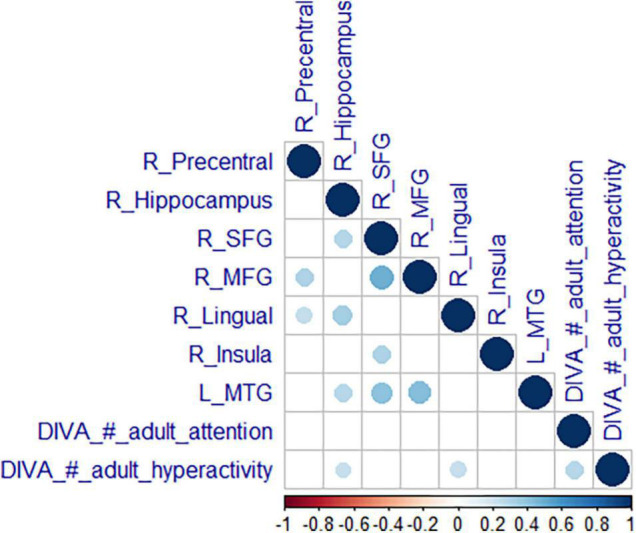
Correlation of neural activity with symptoms. Correlation matrix of mean estimates in the significant clusters from the reactive aggression whole-brain analysis with expressions of the core symptoms (attention and hyperactivity). We are reporting Spearman’s correlation coefficient *p* (bottom scale) with a blue color for a positive and a red color for a negative correlation. Results were Bonferroni corrected.

Additionally, the number of hyperactivity/impulsivity symptoms was correlated positively with the activity in two clusters associated with reactive aggression in the ADHD group; the more hyperactivity symptoms the adults with ADHD had, the more activation was seen in the right hippocampus (Spearman’s ρ = 0.29, *p* = 0.046) and the lingual gyrus (Spearman’s ρ = 0.31, *p* = 0.041). No correlations with neural activation in areas linked to reactive aggression were observed for inattention symptoms, see Figure 4.

The sensitivity analysis of medication effects yielded no significant medication effects, see the section Supplementary Analysis.

## Discussion

In this study, we aimed to identify neural correlates of reactive aggression, in adults with ADHD. To our knowledge, this is the first study to use a behavioral measure of reactive aggression as a marker of ED during implicit emotion regulation in adults with ADHD. We found areas of differential brain activity during emotion processing in the ADHD group in covariance with high reactive aggression to be localized in the limbic system and insula as well as in middle and superior frontal areas.

### Reactive Aggression

In line with previous literature on reactive aggression in ADHD, our analysis of reactive aggression indicated significantly elevated levels of reactive aggression in adults with ADHD compared to the control group (32). Higher scores of reactive aggression were also associated with male sex, in congruence with literature on male reactive aggression and externalizing behavior (5, 10). Previous literature shows that increased reactive aggression often correlates with, or has predictive value for ADHD symptom severity (9), with closer developmental coupling with hyperactivity/impulsivity symptoms (42). Indeed, the association of reactive aggression with the subscales of the DIVA diagnostic instrument used in our study confirmed that symptoms of hyperactivity/impulsivity, but not inattention, were associated with reactive aggression. Interestingly, individuals reporting problems in relationships and/or family or with their self-image showed higher levels of reactive aggression, implying that impairments in the social life-domains could be particularly frequent in people with ADHD and high reactive aggression traits. This finding is in line with the literature on ED being an important predictor for the social, functional, and occupational outcome of ADHD (43, 44). Especially for the persistent phenotype of ADHD, ED such as reactive aggression is considered a constitutional component (45), showing most pronounced expression and associated impairments in adulthood (46). This clinical subgroup might thus be more vulnerable to social impairments and is particularly important to investigate further.

### Functional Magnetic Resonance Imaging Task Activation

The fMRI whole-brain analysis of emotional faces revealed activation in the limbic system (associated with emotion processing and memory), the fusiform gyrus (associated with face processing), broad temporoparietal and frontal networks. These findings are in line with the original paradigm (38), which used this task to investigate manipulations of amygdala responsiveness, as well as a meta-analysis of implicit facial emotion processing (47). Notably, the attention distraction task withdrew attention from the emotional stimulus toward the red dot, to measure implicit ED. This might skew the findings toward the study of emotion hyperreactivity and underrepresent the contribution of impaired executive functioning to the production of emotionally dysregulated behavior in ADHD. Studies on explicit emotion regulation, report more commonly associated areas such as the anterior cingulate and dorsal and ventromedial prefrontal cortex (27), which might be more relevant for tasks without distraction from the emotional stimulus. However, we find evidence, that even in this implicit emotion regulation scenario, middle and superior frontal areas are engaged, suggesting that executive functioning plays a role for implicit emotion regulation as well.

We found no neural activation differences between adults with and without ADHD, irrespective of emotional valence, suggesting that implicit emotional reactivity is not altered in people with ADHD. While several fMRI studies in children with ADHD reported group differences in the activity and connectivity of the amygdala, the ventral striatum, and the OFC, these effects were often moderated by medical treatment and were not replicated in adult populations [for a review see Rubia (17)]. In the current study, *post hoc* analyses of medication effects on reactive aggression, symptoms, impairments and the neural correlates of reactive aggression revealed no significant associations (see section Supplementary Analyses).

However, sex seemed to influence emotion processing in the left orbitofrontral and superior frontal cortex. These areas are implicated in cognitive control processes, relevant for downregulation of emotional responses and might play a role for general sex differences in emotional reactions or aggression.

### Neural Correlates of Reactive Aggression

While the neurocognitive architecture of emotion processing does not seem to be altered over the whole ADHD group compared to the controls, we expected the neurocognitive architecture to differ in adults with ADHD and co-occurring reactive aggression. Therefore, we investigated the association of reactive aggression with whole-brain activity in both diagnostic groups. We found a significant interaction between ADHD diagnosis and reactive aggression in areas such as the lingual gyrus, caudate, superior parietal, middle temporal, and premotor areas during processing of emotional faces, pointing toward differential neural correlates in adults with ADHD and reactive aggression compared to control subjects with reactive aggression.

When focusing the analysis on the ADHD group, activity in the right precentral, right lingual and left middle temporal gyri was particularly increased in people with ADHD if they had higher reactive aggression scores. Furthermore, small clusters within the right insula, the right limbic system (hippocampus and subthreshold parts of the amygdala) and middle and superior frontal areas were implicated in emotion processing in the ADHD group with high reactive aggression only.

The insula as well as the hippocampus and amygdala are associated with the reactivity and assignment of salience during emotion processing in ADHD (31, 48). Emotional reactivity in the amygdala might be influenced by the hippocampus, which controls emotional memory recalling and regulation, especially in positive contexts (49), and the insula, engaged in down-regulation and maintaining homeostasis during the experience of negative emotions (48, 50). Knowing that measures of structure and volume of the brain in these areas show alterations in children with ADHD, one could hypothesize that an altered neurodevelopment of these structures could influence the vulnerability of individuals with ADHD to develop reactive aggressive behavior: differential maturation might lead to higher liability to develop a hyperreactivity of the limbic system, which might suggest a more intense emotional sensation and higher sensitivity to emotional stimuli in subjects with ADHD.

Our results on altered activity of limbic structures and the insula during emotion processing are in line with previous findings in medication-naive individuals with ADHD (22, 51). These authors discuss medication might drive a normalization effect of differential amygdala response. Interestingly, in our sample medication was not associated with reactive aggression, brain activity or ADHD symptoms in adulthood, which suggests that for the persistent phenotype of ADHD in our sample, stimulant medication seemed to be inefficient to further improve symptoms significantly.

Interestingly, we observed higher activity in middle and superior frontal clusters in patients with high reactive aggression scores. This activation is associated with stronger cognitive control and might suggest more effortful top–down emotion regulation, a process frequently disturbed in ADHD (52), implying both subprocesses, emotional reactivity as well as top–down regulation are implied in reactive aggression in adult ADHD.

The activation of the left MTG as well as premotor areas was observed specifically in the ADHD group. The left MTG or temporo-parietal junction is often implicated in theory of mind abilities (53). This ability to understand other people’s beliefs and intentions might represent a protective mechanism for ED. The activity in the MTG could reflect higher efforts to retrieve intentions from the facial expressions in the ADHD group. Higher premotor activity could be related to more allocation of attention. Both could be cause or consequence of higher estimation of salience in the ADHD group with higher reactive aggression scores (e.g., hippocampus), which could trigger a deeper processing of the seemingly important emotional stimuli.

The healthy control group did not show any clusters associated with high reactive aggression, in coherence with the notably low levels of reactive aggression in this group. Interestingly, decreased activity in people in the control group with particularly low reactive aggression scores in some clusters that are implicated in the ADHD group as well suggest a protective function of these areas to develop reactive aggression (in particular the left MTG, precentral, and lingual gyri).

### Associations of Clinical Measures

We found positive correlations between the expression of hyperactivity/impulsivity symptoms and the activity related to reactive aggression in the hippocampus as well as the lingual gyrus in individuals with ADHD. The more hyperactivity/impulsivity symptoms adults with ADHD showed, the more these areas were engaged during emotion processing in association with reactive aggression. This association of ADHD symptoms with brain activity related to reactive aggression could be an example of a deviant neurocognitive mechanism behind ED in ADHD where the brain mediates both, ED as well as ADHD symptoms. Inattentive symptoms or impairments were not significantly correlated with neural activities. These findings point toward a specific susceptibility of the hyperactive type of ADHD to exhibit ED such as reactive aggression, in line with findings on the close developmental coupling of hyperactive/impulsive symptoms with reactive aggression (42) and general association of the hyperactive subtype with ED and aggression (54).

### Strengths and Weaknesses

All results need to be viewed in the context of the strengths and limitations of this study. Our sample sizes exceed most previous fMRI studies related to this topic in ADHD and is demographically well balanced.

However, task activation maps across all subjects did not show some areas classically associated with emotion regulation, such as the anterior cingulate, OFC, dlPFC, and vmPFC, or the amygdala (55), the latter only showing up as small subthreshold clusters. This was likely due to the implicit nature of our task. While emotion regulation paradigms mostly direct the participant’s attention toward the processing of an emotion or even ask for explicit emotion regulation (55), we employed an implicit paradigm in which the participant’s attention was not focused on the processing of emotional content and they were not asked to regulate their emotions, potentially affecting effect sizes and activity in areas relevant for top–down control. Notably, the clustersize of the reported results of the covariance of reactive aggression with ADHD is overall small, all described results should be carefully discussed as true findings. The small clustersizes might be related to the small effect sizes elicited from implicit emotion processing task in combination with an indirect measure of trait-reactive aggression as well as the vast heterogeneity in the ADHD population. Future studies with sufficient statistical power to address for expected small effect sizes that investigate subgroups within the spectrum of ADHD could elucidate this matter further. Furthermore, contrasting the emotional conditions to a fixation cross (implicit baseline) instead of neutral faces, as we did here, expectedly captured not only emotion processing but also general social cognition processes. For the investigation of adult ADHD and the association to ED, broader social processes, e.g., general face processing difficulties, might play a role as well. Notably, we included reactive aggression as a trait-behavioral measure of ED. The applied task is measuring emotion processing closer to everyday-life situations compared to explicit emotion regulation tasks. Importantly, the integration of trait behavioral reactive aggression as a reflection of very relevant real-life ED together with the activity during emotion processing is suitable to reveal which areas are relevant for emotionally dysregulated behavior and potentially highlight social cognition and emotional (sub)processes that are implicated in ED. However, the implementation of a task to elicit an emotional response, without attention distraction during fMRI could result in bigger effect sizes and a more precise delineation of the network of ED in ADHD. We furthermore recommend the implementation of a longitudinal design to study relevant brain networks for ED in ADHD, due to the close coupling of developmental trajectory of hyperactivity/impulsivity with the expression of ED.

## Conclusion

In conclusion, these findings convey evidence for a differential emotion processing mechanism in subjects with ADHD and reactive aggression, with a specific liability of individuals with hyperactivity/impulsivity symptoms to experience these alterations. The brain regions related to this mechanism suggest difficulties with both emotional hyper-reactivity (as reflected in the insula and amygdala), and more effortful regulation of emotional responses (implicated by hippocampus and frontal activity) in the ADHD group with higher reactive aggression. This differential mechanism appears to be related to an altered neurocognitive brain architecture of ADHD and supports the diagnostic and clinical view of emotionally dysregulated ADHD as a subgroup in the spectrum of the disorder, as discussed by Shaw P et al. (3). In the light of these findings, future research may evaluate more targeted intervention for the emotionally dysregulated group, such as behavioral emotion regulation interventions and their effect on reactive aggressive behavior (56).

## Data Availability Statement

The raw data supporting the conclusions of this article will be made available by the authors upon motivated request with the intention to investigate ADHD.

## Ethics Statement

The studies involving human participants were reviewed and approved by CCMO. The patients/participants provided their written informed consent to participate in this study.

## Author Contributions

MH, BF, and AA-V contributed to study conception and supervision, obtained the funding. PV and BJ provided the samples and data. BJ and DR conducted the analyses and data interpretation. BJ, DR, MH, and AA-V contributed to writing group. All authors contributed to manuscript revision, read and approved the submitted version.

## Conflict of Interest

The authors declare that the research was conducted in the absence of any commercial or financial relationships that could be construed as a potential conflict of interest.

## Publisher’s Note

All claims expressed in this article are solely those of the authors and do not necessarily represent those of their affiliated organizations, or those of the publisher, the editors and the reviewers. Any product that may be evaluated in this article, or claim that may be made by its manufacturer, is not guaranteed or endorsed by the publisher.
