# Supplementary material for: Neural Correlates of Reactive Aggression in Adult Attention-Deficit/Hyperactivity Disorder
Source: Front Psychiatry. 2022 May 19;13:840095. doi: 10.3389/fpsyt.2022.840095 (PMC9160326; doi:10.3389/fpsyt.2022.840095)
Supplement: Supplementary file 1 [file Data_Sheet_1.pdf]

Supplementary Material

1 Figures

1.1 Figure SF1. Example of the presented stimuli.

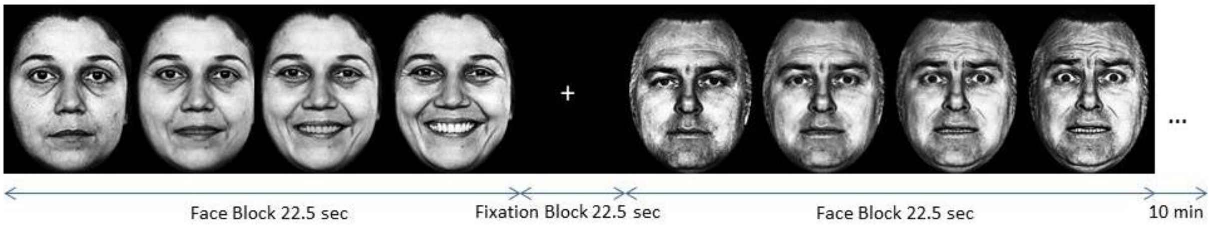

Supplementary Figure SF1. Example of the presented stimuli. Schematic example of a trials with a female actor in a happy face block and a male actor in a fear face block. Faces morphing from a neutral expression to the target expression in 450ms, described here in four example frames.

1.2 Figure SF2. Overlap of emotion processing networks.

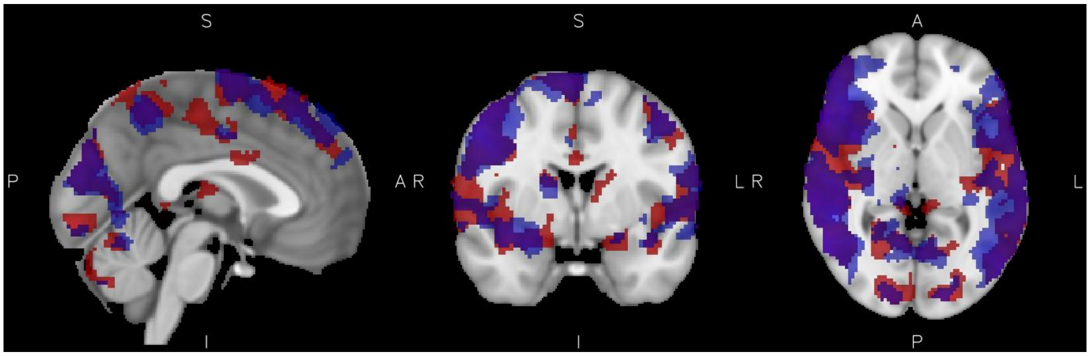

Supplementary Figure SF2. Overlap of emotion processing networks. Results from the whole-brain contrast analysis Emotion<Fixation for the ADHD group in red overlapped with the control group in blue. Results are FWE-corrected at a significance level of $p<0.05$ at xyz = (0,0,0).

2 Tables

2.1 Table ST1. Broad-scale emotion processing networks.

Cluster index	Voxels	P	Z-MAX	X (mm)	Y (mm)	Z (mm)	Region of the Peak

**ADHD**							
5	51174	< 0.001	11,40	62	-24	18	Parietal Operculum
4	2263	< 0.001	9,83	16	-94	0	Occipital Pole
3	199	< 0.001	5,43	24	-78	-36	Cerebellum
2	194	< 0.001	5,53	-16	-10	20	Left Caudate
1	130	0,0385	5,16	-20	2	-16	Left Parahippocampal Gyrus / Left Amygdala
**Control**							
3	56835	< 0.001	10.6	52	-38	8	Supramarginal Gyrus
2	572	< 0.001	5.57	-14	-92	-4	Occipital Pole
1	560	< 0.001	5.52	-22	54	16	Frontal Pole

Supplementary Table ST1. Broad-scale emotion processing networks. Results of the whole-brain analysis for control subjects for the contrast Emotion < Fixation family-wise error corrected at p = 0.05. Cluster indices are reported for big cluster spanning several areas, the region of the peak according to the Harvard-Oxford cortical (and if relevant subcortical) atlases are indicated.

2.2 Table ST2. Regression of Proactive Aggression

Regressors	Estimate	Standard Error	t-value	p-value
ADHD diagnosis (y/n)	0.11	0.38	1.53	0.13
Age	-0.13	0.02	-1.76	0.08
Sex	-0.31	0.39	-4.03	<0.001***

Table ST2. Regression of proactive aggression. Summary of regression analysis of proactive aggressive behavior, showing regression coefficients, standard errors, t- and p-values as well as levels of significance in codes from 0.001 as '***' , from 0.01 as '**' or from 0.05 as '*'.

2.3 Table ST3. Effects of Sex on emotion processing

Cluster index	Voxels	P	Z-MAX	X (mm)	Y (mm)	Z (mm)	Region of the Peak
1	36	< 0.05	3,58	-12	16	62	Left SFG
2	33	< 0.05	3,64	-48	36	-14	Left Orbital Frontal Cortex

Table ST3. Effects of sex on emotion processing. Results of the whole-brain analysis for Sex effects during the fMRI paradigm, cluster extent correction of 11 voxel for p =0 .05

3 Supplementary Analyses

3.1 Sensitivity Analysis

Our participants with ADHD were asked to withhold medication for at least 24 hours prior to participation, but long term stimulant treatment as well as therapeutic interventions could drive a generalized normalization effect of the limbic activity. In a post-hoc multivariate linear model we checked if ongoing treatment with stimulants was associated with I) reactive aggression scores, II) the number of inattentive symptoms, III) the number of hyperactive/impulsive symptoms, IV) impairments of the five life domains of the DIVA and V) the activity in the clusters from the reactive aggression analysis. We found no significant associations of medication with either variable.

3.2 Analysis of proactive aggression

While proactive aggression was not associated with ADHD diagnosis, it was significantly associated with male sex, which is in line with research reporting elevated levels of aggression in males.
